# Research on High Temperature Stamping Forming Performance and Process Parameters Optimization of 7075 Aluminum Alloy

**DOI:** 10.3390/ma14195485

**Published:** 2021-09-22

**Authors:** Zheng Ma, Hongchao Ji, Xiaomin Huang, Wenchao Xiao, Xuefeng Tang

**Affiliations:** 1 College of Mechanical Engineering, North China University of Science and Technology, Tangshan 063210, China; 18131736601@163.com; 2 School of Mechanical Engineering, University of Science and Technology Beijing, Beijing 100083, China; hxm8606@126.com; 3 School of Engineering and Technology, China University of Geosciences (Beijing), Beijing 100083, China; xwcxiaowenchao@163.com; 4 State Key Laboratory of Materials Processing and Die & Mould Technology, Huazhong University of Science and Technology, 1037 Luoyu Road, Wuhan 430074, China; xftang@hust.edu.cn

**Keywords:** 7075 aluminum alloy, forming limit diagram, GA-BP neural network, automotive S-rail, process parameters

## Abstract

The stress strain curve of 7075 aluminum alloy in the temperature range of 310 °C to 410 °C was obtained by Gleeble-3800. By Nakazima test, the isothermal thermoforming limit diagrams of 7075 aluminum alloy at different deformation temperatures and stamping speeds were acquired. Moreover, the parameters of automotive S-rail hot stamping process were optimized by GA-BP neural network. The results show that the forming limit curve of 7075 aluminum alloy increases as the deformation temperature and stamping speed increase. The predicted optimal parameters for hot stamping of automotive S-rails by GA-BP neural network are: stamping speed is 50 mm/s, friction coefficient between die and blank is 0.1, and blank holder force is 5 kN. The maximum thinning rate at this process parameter is 9.37%, which provided a reference for 7075 aluminum alloy automotive S-rail hot stamping.

## 1. Introduction

Due to severe environmental pressures and the increasing depletion of energy, higher requirements have been placed on the automobile manufacturing industry. The trends of today’s automotive industry are energy saving, environmental protection and safety. Under the premise of ensuring the safety of the car, reducing the weight of vehicles can effectively achieve energy savings and reduce emissions [1,2]. The advantages of high strength, recyclability and good performance make aluminum alloy one of the most ideal materials in current automotive lightweighting technology [3]. However, the plastic elongation of aluminum alloy materials is low at room temperature, therefore, most aluminum alloy sheet metal parts are formed by hot stamping [4].

The process of aluminum alloy hot stamping is: first, the high-strength light alloy sheet such as aluminum alloy is heated, generally to 300 °C~500 °C [5,6], so that the alloy material has good elongation. Then, the heated alloy sheet is quickly moved to the forming die device for stamping, so as to obtain a light alloy structure with good formability, smaller rebound and higher strength.

One of the important goals of the sheet metal forming process design is to manufacture the blank into the required shape without any failures, such as fracture and excessive local thinning. The determination and prediction of the forming limit are of great significance to prevent material failures in sheet metal forming. Bruschi et al. [7] reviewed the development of testing methods and modeling theory in the field of sheet metal forming. Among them, the forming limit diagram (FLD) [8] is the most widely used and direct method in the industry. Killer et al. [9,10] first proposed the concept of forming limit diagram, which was widely used to predict the comprehensive forming performance of sheet metal. Currently, there are two typical methods to determine FLD: the Nakajima test [11] and the Marciniak test [12]. Generally, the FLD can completely cover various strain paths that appear in industrial forming operations. In order to achieve different strain paths, loads that generate different strain paths are applied to the sheet specimen, so that uniaxial stretching becomes biaxial stretching.

In recent years, many scholars have conducted research on 7075 aluminum alloy hot stamping. Xiao et al. [13] studied the influences of process parameters such as blank temperature, stamping speed, blank holder force, and friction coefficient on the formability and mechanical properties of 7075 aluminum alloy deep drawing, based on the hot stamping process. Liu et al. [14] studied the formability of 6061 and 7075 aluminum alloy hot stamping automotive B-pillars under different solution heating times and different lubrication conditions. Song et al. [15] proposed a servo motion to study the hot stamping formability of 7075 aluminum alloy thin-walled cylindrical parts, and revealed the law and mechanism of the influence of servo motion on the formability of thin-walled cylindrical parts. Liu et al. [16] developed a novel experimental apparatus for measuring the temperature variation of sheet and die for hot stamping of 7075 aluminum alloy under dry and lubricated conditions with different contact pressures. Rong et al. [17] proposed an improved continuous damage model and incorporated it into a set of uniaxial unified viscoplastic constitutive equations to describe the thermal flow behavior of AA7075 alloy. Then, the damage correction formula was introduced into the damage model to predict the hot forming limit diagram of AA7075 at high temperature. Ying et al. [18] determined the thermoforming limit diagram (TFLD) of AA7075 aluminum alloy by experiment and numerical simulation. Liu et al. [19] developed an initial blank temperature correlation model to predict the interfacial heat transfer coefficient (IHTC) of 7075 aluminum alloy at different initial blank temperatures and verified it through the results of non-isothermal forming tests. Gao et al. [6] used the GTN model to predict the mechanical properties of 7075 aluminum alloy thermal in a small punch test experiment. They verified that the GTN damage model can predict the damage evolution of the B-pillar. Xiao et al. [5] predicted the forming limit curve of 7075 aluminum alloy at high temperature based on the continuous damage mechanics model.

In this paper, the high temperature mechanical properties of 7075 aluminum alloy are determined through uniaxial hot tensile experiment. The forming limit diagrams of 7075 aluminum alloy at different temperatures and stamping speeds were obtained by Nakazima test, and the mechanical properties and microstructure of the formed parts were analyzed. In addition, the process parameters of 7075 aluminum alloy automobile S-rail hot stamping forming are optimized through GA-BP neural network and the optimization results are analyzed and verified, which can be used to improve hot stamping formability and reduce forming defects, providing a reference for 7075 aluminum alloy hot stamping forming. The results show that the aluminum hot stamping process can significantly improve the formability without reducing the mechanical properties.

## 2. Materials and Experiment

### 2.1. Material

**Figure 1:**
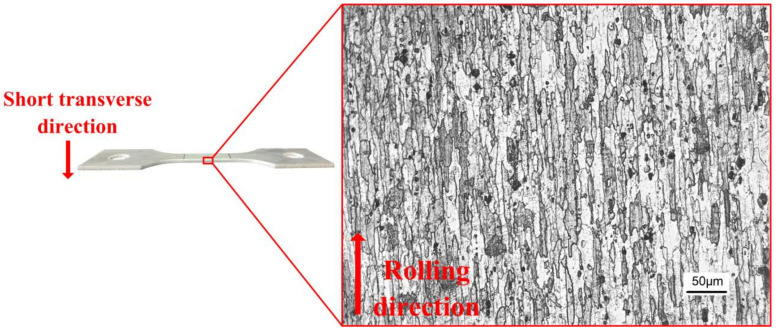
Short transverse microstructure of 7075 aluminum alloy.

In the experiment, rolled 7075 aluminum alloy with the thickness of 2 mm was selected. The chemical composition of the alloy is (wt %) 0.07 Si-0.22 Fe-1.4 Cu-0.01 Mn-2.2 Mg-0.19 Cr-5.4 Zn-0.02 Ti-(bal.) Al. The properties of the materials at room temperature conditions are shown in Table 1 [13]. Figure 1 shows the microstructure along the short transverse (ST) of the rolled alloy. In Figure 1, typical elongated pancake-like particles can be observed, with an average thickness of 12 μm, in which sediments are distributed.

### 2.2. High Temperature Tensile Test

**Figure 2:**
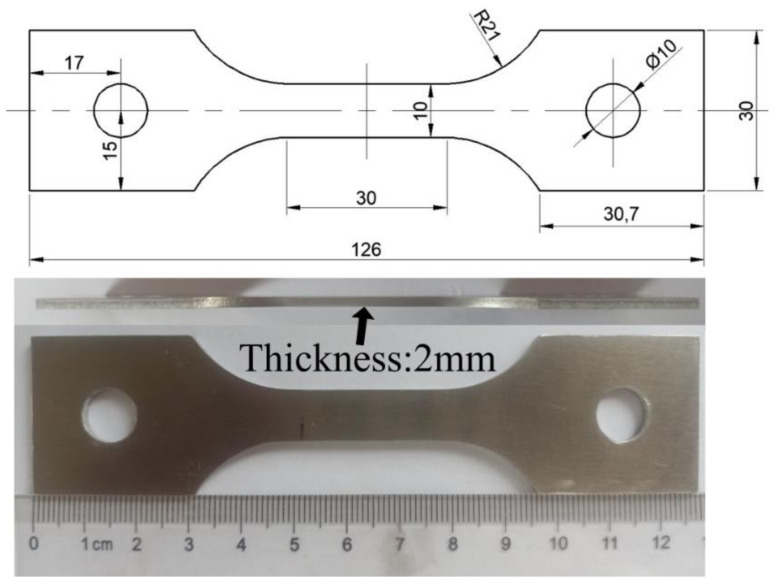
The shape and size of the sample.

To measure the mechanical data of 7075 aluminum alloy under high temperature conditions, the Gleeble-3800 thermal simulation testing machine was used to perform constant high temperature stretching of 7075 aluminum alloy. Gleeble-3800 adopts computer programming control technology and hydraulic power control technology, which has the characteristics of high precision and good repeatability of simulation tests. The Gleeble-3800 selected for this test is manufactured by FULETE Instrument Technology (Shanghai) Company Limited (Shanghai, China). The shape and size of the sample are shown in Figure 2 (unit: mm). A wire cutting machine is used to cut the sheet into a tensile sample. The length of the sample is the same as the rolling direction of the sheet, and the outline of the sample is smoothed with 400# sandpaper.

**Figure 3:**
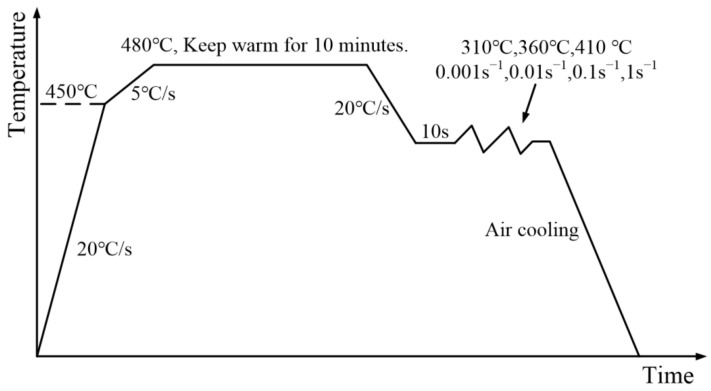
Sample temperature history.

Combined with the hot stamping process, the design of high temperature tensile experiment scheme is shown in Figure 3. First, the sample was heated to 450 °C at a rate of 20 °C/s, and then to a solution temperature of 480 °C at a rate of 5 °C/s, and then kept warm for 10 min. The sample was reduced to the deformation temperature at 20 °C/s, and the temperature was kept for 10 s to make the temperature of the sample uniform. The tensile test was carried out at a constant temperature and strain rate, and the specimen was quickly cooled after being broken to preserve its microstructure at high temperature. The deformation temperature is 310 °C, 360 °C, 410 °C, and the strain rate is 0.001 s^−1^, 0.01 s^−1^, 0.1 s^−1^, 1s^−1^, respectively.

**Figure 4:**
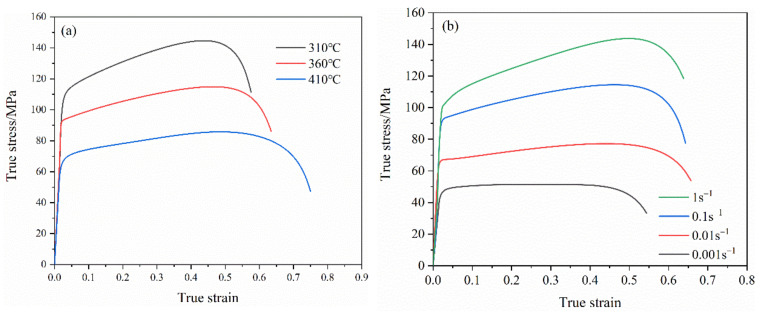
The stress-strain curve of 7075 aluminum alloy under different conditions. (**a**) The strain rate is 0.1, (**b**) the deformation temperature is 360 °C.

Figure 4 shows the true stress-strain curve of 7075 aluminum alloy sheet under different deformation temperatures and different strain rates. It can be seen from the figure that at the same deformation temperature, the flow stress increases significantly with the increase of the strain rate. At the same time, it is also found that at the same deformation temperature, the ultimate strain of the sample increased slightly with the increase of the strain rate. At the same strain rate, the flow stress decreases with the increase of the deformation temperature, which is manifested as thermal softening.

### 2.3. Isothermal Forming Limit Experiment of Aluminum Alloy Sheet

**Figure 5:**
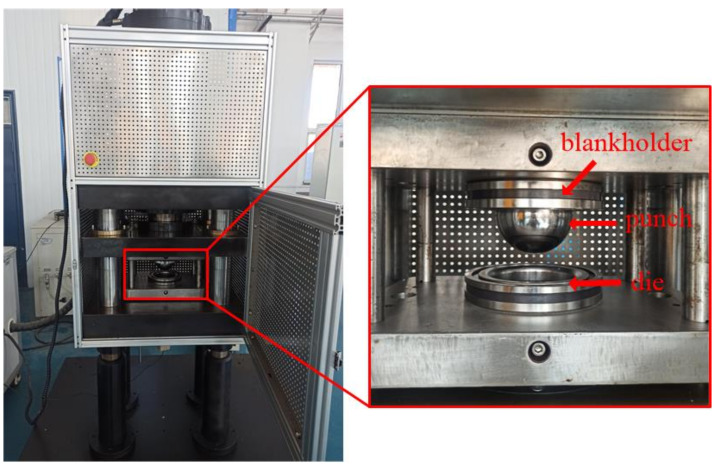
Sheet forming test machine.

**Figure 6:**
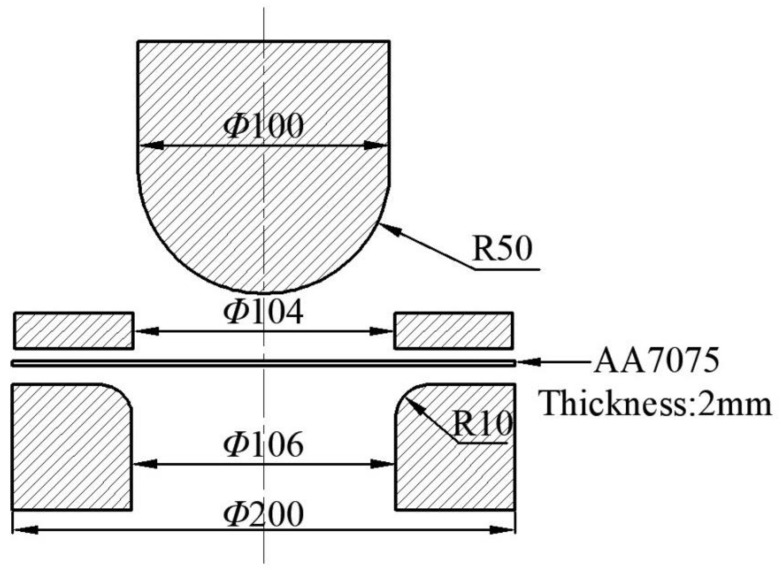
Dimensions of sheet metal forming test machine (unit:mm).

The hemispherical rigid punch bulging test method (Nakazima test) was used to study the isothermal thermal deformation behavior of 7075 aluminum alloy under plane stress. The experimental equipment is the muffle furnace of model SXL-1700C (SHANGHAI JUJING PRECISION INSTRUMENT MANUFACTURING CO., LTD., Shanghai, China) and the sheet forming test machine of model EC600H (SHANGHAI GENBON INDUSTRIAL CO., LTD., Shanghai, China), as shown in Figure 5. The muffle furnace has high temperature control accuracy and good heat preservation effect, and is used for heating blanks. The sheet forming test machine is mainly composed of a punch, a die and a blank holder. The specific dimensions are shown in Figure 6 (unit: mm). During the working process, the blank is placed on the die, and then the blank holder moves downwards to compress it with a constant blank holder force (6 kN), which is provided by a nitrogen gas spring. Subsequently, the punch moves downwards, and the blank is deformed in the process of contact with the punch. Stretching ribs are arranged on the blank holder to prevent the sheet material from flowing into the cavity in the die during the deformation process.

**Figure 7:**
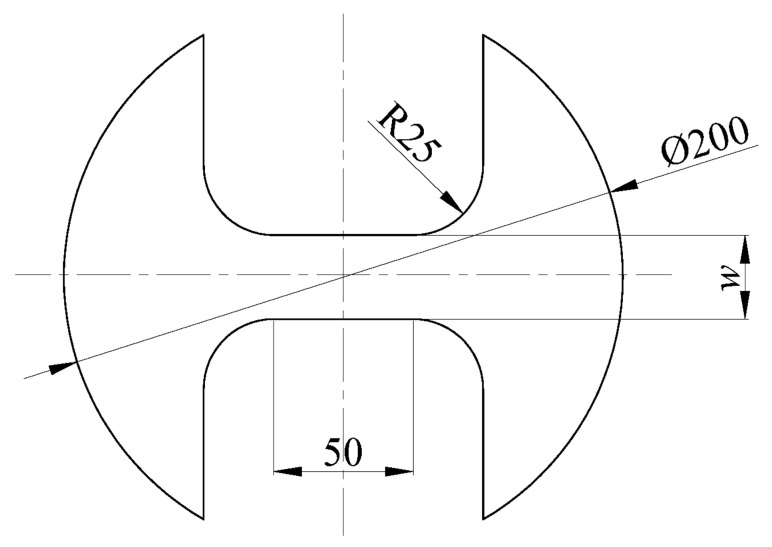
The geometry and size of the blank (unit: mm).

The blank geometry and size are shown in Figure 7. The blank width *w* in the figure corresponds to 5 groups of different sizes, which are 30 mm, 80 mm, 100 mm, 150 mm and 200 mm. The thickness of the blank is 2 mm. Blanks with different widths correspond to different stress states, and the blanks with a width of 30 mm are subjected to uniaxial tensile stress during the deformation process. The blank with a width of 200 mm is subjected to double tensile stress during the deformation process. The rest of the width of the blank is under plane stress during the deformation process, and the stress ratio is between the blank with a width of 30 mm and the blank with a width of 200 mm. The circular grid with the diameter of 2 mm is applied to the surface of the blank by electrochemical corrosion to facilitate subsequent strain measurement. In the process of blank deformation, the experiment will be stopped when the blank is locally necked or just ruptured. The deformation grid near the neck or the fracture site is measured to calculate the local ultimate strain. Then, the ultimate strain under different deformation conditions is plotted on the main strain diagram to form the forming limit curve (FLC).

**Figure 8:**
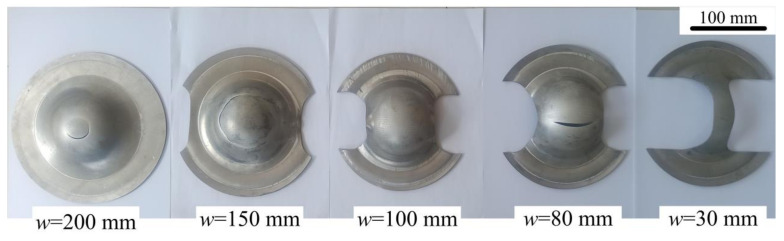
Isothermal forming of a limit fracture of 7075 aluminum alloy blank.

During the experiment, the die is heated to the forming temperature (310 °C, 360 °C, 410 °C) with the heating rod, and the blank is heated to the deformation temperature in the muffle furnace and kept for 10 min. Subsequently, the blank is manually transferred to the sheet forming test machine within 3 s, and the positioning is completed. Taking into account the temperature drop of the blank during the manual transfer process, the heating temperature of the blank should be increased by 10 °C compared with the deformation temperature to ensure that the temperature of the blank is consistent with the die temperature and achieve isothermal forming. The central area of the blank (diameter <100 mm) and the punch are fully lubricated with dry graphite lubricant, and the contact friction coefficient between the punch and the blank is about 0.1 [20]. The movement speed of the hydraulic press is controlled to adjust the punching speed. The adjustable range of the punching speed is about 10–40 mm/s, and the corresponding strain rate is 0.2–0.8 [21]. Each experiment is repeated 3 times to ensure the accuracy of the experiment. Figure 8 shows part of the 7075 aluminum alloy blank isothermal forming a limit fracture.

After the completion of the experiment, it is necessary to select the critical grid used to calculate the strain, namely, the largest deformed grid next to the broken grid. The measured grid size is converted into the maximum and minimum strain, which are projected in the coordinate system to obtain the forming limit diagram at the corresponding process conditions [22,23].

## 3. 7075 Aluminum Alloy Isothermal Forming Limit Analysis

### 3.1. Results of Forming Limit Diagram

**Figure 9:**
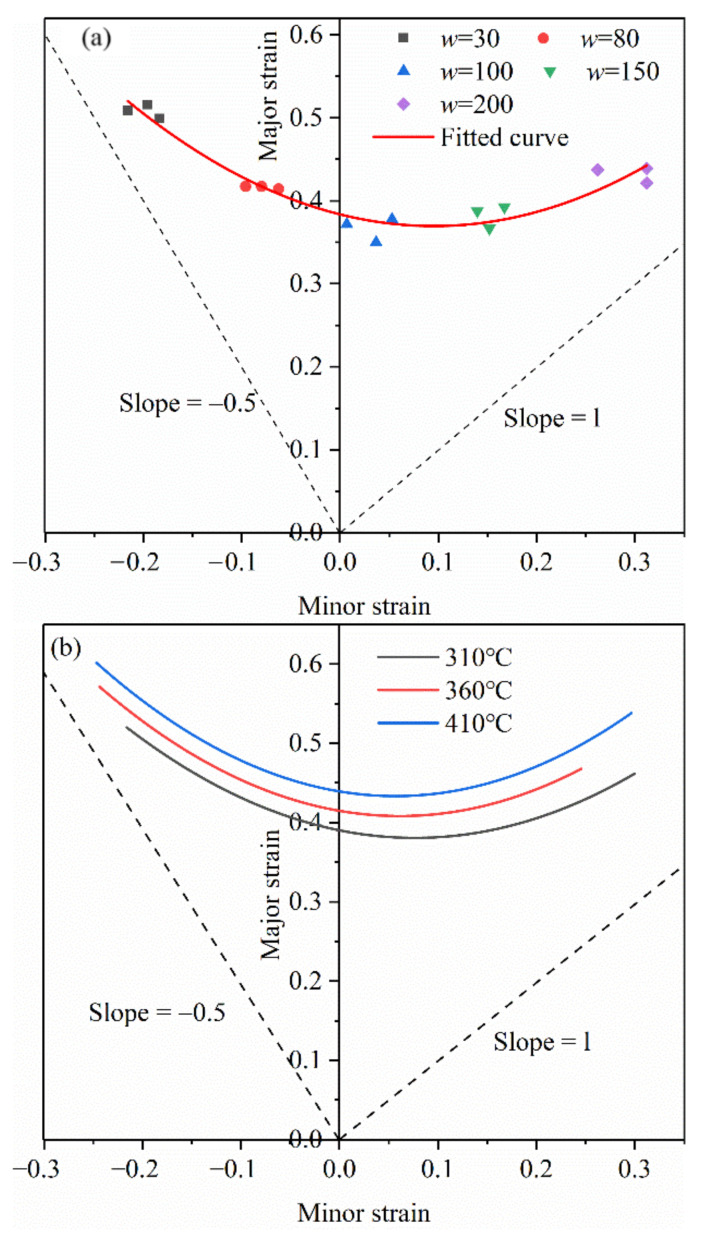
(**a**) 360 °C forming limit diagram, (**b**) forming limit curves at different temperatures.

Figure 9a is the forming limit diagram of 7075 aluminum alloy when the stamping speed is 20 mm/s and the forming temperature is 360 °C. It can be seen from the figure that the maximum principal strain of the uniaxial stretching zone is greater than the maximum principal strain of the biaxial stretching zone, which make the FLC present an upward warping trend on the left. If the principal strain is below 0.408, the effectiveness of the formed part can be guaranteed. If the principal strain is higher than this value, the formed part is prone to cracking failure. Figure 9b shows the forming limit diagram at the stamping speed of 20 mm/s and different deformation temperatures. It can be seen from the figure that the forming limit curve of 7075 aluminum alloy has improved as the temperature increases, which means that the forming limit of the material has increased. In the plane strain state, the maximum principal strain increased from 0.381 at 310 °C to 0.434 at 410 °C, an increase of about 14%.

**Figure 10:**
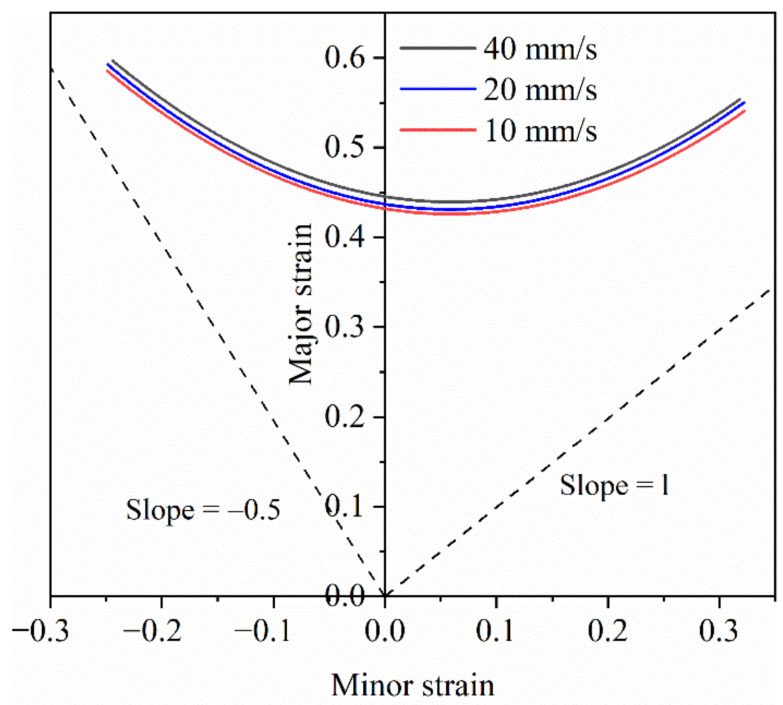
Forming limit curves at different stamping speeds of 410 °C.

Figure 10 shows the effect of stamping speed on the forming limit curve of 7075 aluminum alloy when the deformation temperature is 410 °C. It can be seen from the figure that when the stamping speed increases from 10 m/s to 40 mm/s, the forming limit of the material is slightly increased, and the change is not very obvious. The specific reason is that in the isothermal Nakazima test, the strain rate corresponding to the punching speed of 10 mm/s to 40 mm/s is 0.2–0.8, and the strain rate in the experiment is only expanded by 4 times. Therefore, the impact of the stamping speed on the forming limit is very small, only increasing by 2%. It can be considered that when the stamping speed is within 10 mm/s to 40 mm/s, the impact of stamping speed on the forming limit is negligible, and the change of stamping speed has basically no effect on the forming limit of 7075 aluminum alloy.

### 3.2. Fracture Morphology Analysis

**Figure 11:**
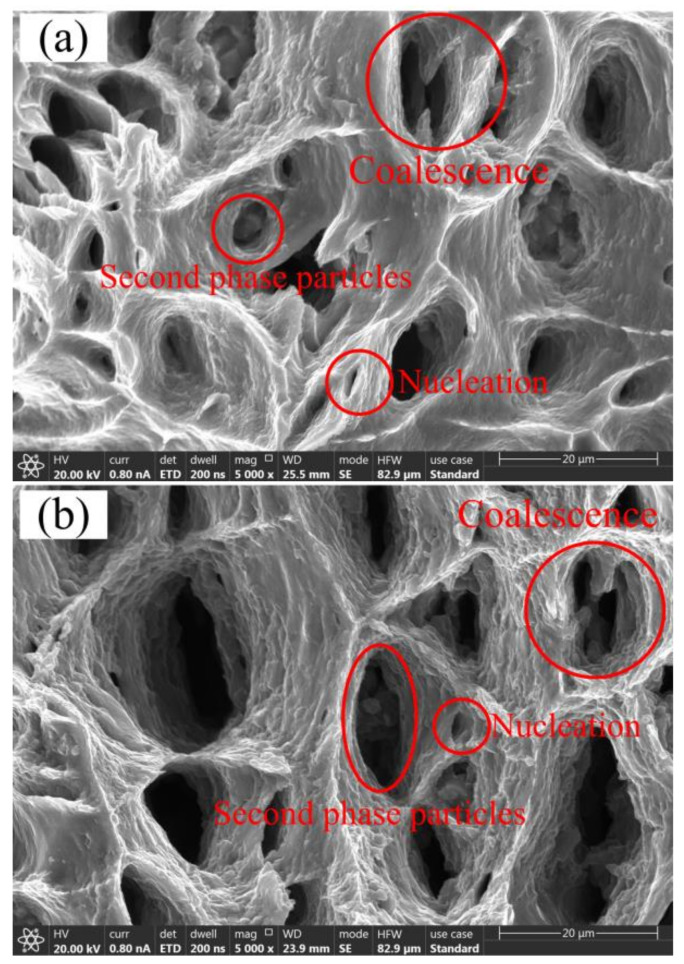
Fracture morphology of *w* = 30 (as shown in Figure 7) samples at different temperatures. (**a**) 310 °C, (**b**) 360 °C.

A scanning electron microscope (SEM, Shanghai Opton Instrument Co., Ltd., Shanghai, China) was used to observe the fracture morphology of the samples at different conditions. Figure 11 shows the fracture morphology of the sample with a width of 30 mm at 310 °C and 360 °C. It can be seen from the figure that there are various dimples and voids distributed in the fracture of the sample at 310 °C and 360 °C. The number and size of the dimples are related to the number and size of the second phase in the material [24,25]. When the width is 30mm, the strain path is approximately uniaxial stretching, so the dimple shape is similar to the dimple shape in uniaxial thermal stretching. Dimples appear when the material fractures by ductile fracture [26]. When the deformation temperature is 360 °C, the number of dimples is less than that at 310 °C, but the depth and size of the dimples increases. The depth of the dimple is consistent with the plasticity of the material, and there is a deeper dimple at 360 °C, indicating that the sample has better plasticity at this time, which is consistent with the result of Figure 9b. It can be seen from the figure that there are obvious second-phase particles in some dimples, which indicates that some voids nucleate at the second-phase particles. In addition, the nucleation and coalescence of micro-voids can be seen in the figure. The micro-voids nucleate, grow and accumulate into larger voids, thereby generating cracks.

**Figure 12:**
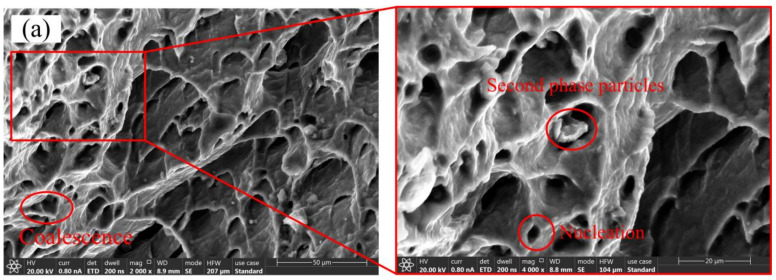

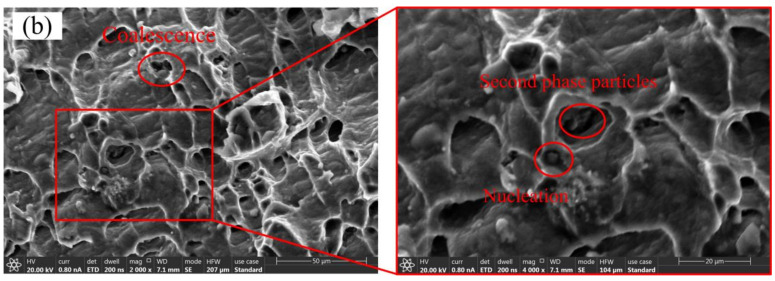
Fracture morphology of samples with different widths at a deformation temperature of 360 °C (**a**) *w* = 80, (**b**) *w* = 200 (as shown in Figure 7).

Figure 12 shows the fracture morphology of the sample with a width of 80 mm and 200 mm at a deformation temperature of 360 °C. From the figure, we can see the obvious second phase particles, the nucleation and coalescence of the voids. In addition, the dimples of the sample with a width of 80 mm are flat, and the dimples of the sample with a width of 200 mm are regular and circular. This is because the sample with a width of 200 mm is in a biaxial tension state, and the force received by the sample in the vertical direction in the plane is equal. The sample with a width of 80 mm is in the state of plane edge response. The force on the specimen in the vertical direction in the plane is not equal, and the dimple is elongated in the direction where the sample receives the greatest force. At the same time, it can be seen in the figure that as the width of the sample increases, the number of dimples and the size of the dimples decreases significantly. This shows that the sample with a width of 200 mm has better plasticity, which is consistent with the result in Figure 9a.

## 4. Optimization of Hot Stamping Process Parameters of Automobile S-Rail

### 4.1. Finite Element Model

**Figure 13:**
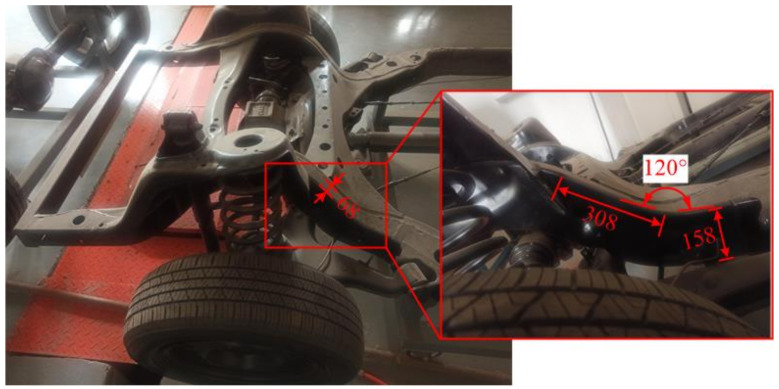
Automobile S-rail (unit:mm).

There are many car body parts and using aluminum alloy to make car body products can reduce the weight of the car body to achieve the goal of a lightweight car. The requirements for stamping and forming technology have also been continuously improved. The S rail is a component that absorbs energy inside the car body when the car collides. Therefore, it needs to be strong. It is divided into an axial rail and a lateral rail (S-shaped rail). Its position on the car body is shown in Figure 13.

**Figure 14:**
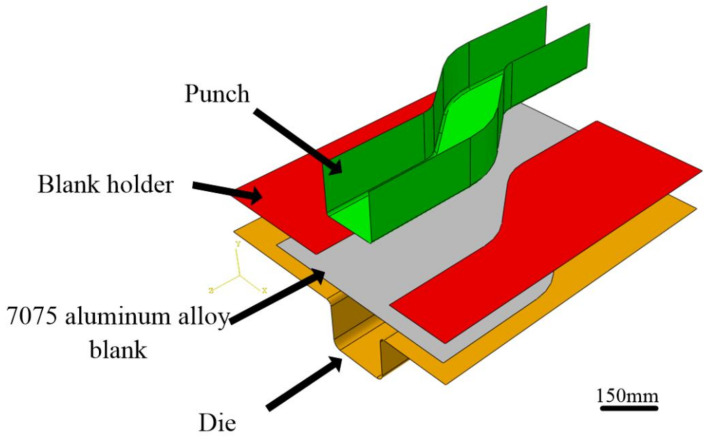
Finite element model of automobile S-rail hot stamping.

The stamping dies and blank of the automobile S-rail were drawn in CREO software, as shown in Figure 14. The dies include punch, blank holder and lower die. In the ABAQUS software, in order to save analysis time, the dies and the blank choose the shell. The dies were set as the rigid body. The blank was set as a deformable shell, and the initial thickness of the blank was set to 1 mm. The basic mechanical properties of 7075 aluminum alloy, such as density, elastic modulus and Poisson’s ratio were inputted in the material properties. According to Figure 4, we inputted the plastic mechanical properties at different temperatures and strain rates, as well as the forming limit diagram obtained in the upper part into the FLD damage in ABAQUS material properties. The thermal properties of 7075 aluminum alloy are shown in Table 2 [18]. The analysis step of the model is a thermal-mechanical coupling analysis step. The die was set to room temperature (25 °C) and the sheet material was set to the forming temperature. The heat transfer between the tool and the blank was also considered. If the distance between the die and the blank exceeds 2 mm, it was ignored. Otherwise, it was set to a constant value of 16 kW·m^−2^·°C^−1^ [27].

The hot stamping process is: (1) Fix the die. (2) Apply a blank holder force to the blank holder. (3) Punch moves down 40 mm at a constant speed.

### 4.2. Orthogonal Experimental Design Based on Hot Stamping Process

An orthogonal experiment is a design method that studies multiple factors and multiple levels [28,29]. It selects a representative combination of factors from all the factor level combinations of the comprehensive experiment to conduct the experiment, which can greatly reduce the workload. By analyzing these representative combinations, useful and objective conclusions can be obtained efficiently and quickly. Therefore, orthogonal experiments have been widely used in scientific research in various fields [30,31].

The process parameters of hot stamping mainly include blank holder force, blank temperature, stamping speed, friction coefficient between blank and die, die gap and so on. The process parameters have a great influence on the performance of the sheet metal after forming. Since the optimal forming temperature of 7075 aluminum alloy is about 400 °C [32], the blank temperature selected in this article is 410 °C. Numerical simulation is used to study the influence of blank holder force, stamping speed and friction coefficient between blank and die on the maximum thinning rate after blank forming. Therefore, an orthogonal test with three factors and three levels is established. Table 3 is the level table of the orthogonal experiment of the numerical simulation.

According to the well-divided factors and levels in Table 3, a three-factor and three-level orthogonal experimental design was constructed. The selected orthogonal table is L_27_(3^3^). Through numerical simulation, the orthogonal test scheme of the maximum thinning rate based on the hot stamping process parameters was obtained, as shown in Table 4.

**Figure 15:**
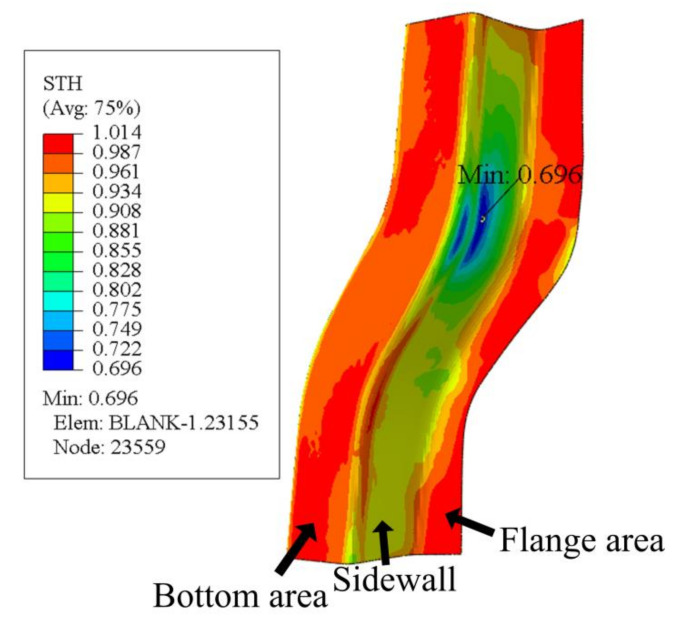
Thickness cloud map at stamping speed of 10 mm/s, friction coefficient of 0.1, and blank holder force of 10.5 kN, forming temperature 410 °C.

The cloud map of sheet thickness under some conditions is shown in Figure 15.

### 4.3. Establishment of GA-BP Network

Back Propagation (BP) neural network is a multi-layer feedforward network trained according to the error back propagation algorithm. The basic idea of BP neural network is the gradient descent method, which uses error back propagation and repeated learning and training to minimize the mean square error between the actual output value and the expected output value of the network. Neural network is not only the basis of parallel processing and large-scale computing, but also a highly nonlinear dynamic and adaptive organizational system. BP neural networks can be used to describe cognition, decision-making and control intelligent behavior [33,34], which has non-linear mapping capabilities and is good at finding rules from input and output signals. It does not require precise mathematical models and has strong parallel computing capabilities. It is easy to perform programming calculations of software and hardware [35].

**Figure 16:**
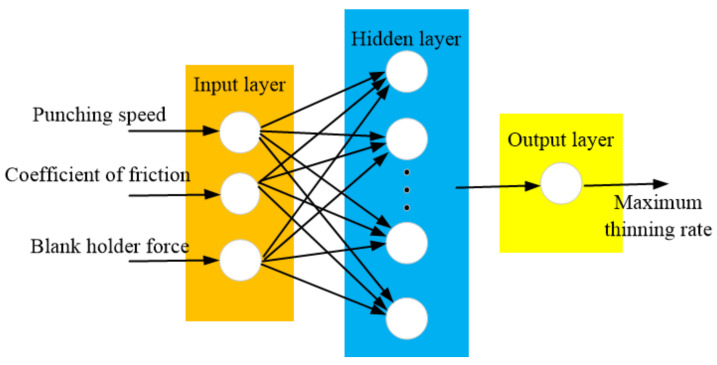
BP neural network structure.

BP neural network is a three-layer feedforward neural network (input layer, hidden layer, output layer), as shown in Figure 16. Forward propagation and backward error transfer are completed by the learning process of BP neural network. First, in the forward propagation process, the input information will be processed by the hidden layer and passed to the output layer. The state of neurons in each layer only affects the next layer. If the desired result cannot be obtained, the error change of the output should be calculated. Then, in the reverse error transfer process, the error between the actual value and the output will return along the previous joint access, and change the joint weight to reduce the error. Finally, it returns to the forward propagation and calculates repeatedly until the error is less than the set value [36,37].

In this paper, a three-layer BP network model was chosen. In nonlinear function fitting, there are 3 input parameters and 1 output parameter. The hidden layer selects 7 nodes, which are calculated according to the formula of Equation (1).

(1) $$m=\sqrt{n+l}+\alpha$$

where: *n* is the input nodes number. *l* is the output node. α is a constant and the range of α is 1–10. The Sigmoid function is chosen for the transfer function, which is:

(2) $$f(x)=\frac{1}{1+e^{-x}}$$

In applications, BP neural networks have limitations in dealing with the problem. BP neural networks use the steepest descent method, which has the advantages of simplicity, a small amount of calculation and strong parallelism. However, it has the disadvantages of slow convergence in the learning process, susceptibility to falling into a local minimum, an incomplete algorithm, poor robustness and poor network performance [38]. To address the limitations of BP networks, there are three approaches: (1) additional momentum [39], (2) adaptive learning rate method, (3) genetic algorithm [40]. Genetic algorithms are metaheuristic algorithms inspired by natural selection processes and belong to the broad category of evolutionary algorithms. Genetic algorithms typically rely on biologically inspired operators such as variation, crossover and selection to generate high-quality solutions to optimize and search for problems [41,42]. Its main advantages are robustness, simplicity and generality. Therefore, BP neural networks are combined with genetic algorithms to optimize the weights and thresholds of neural networks and then use BP neural networks to solve the problem.

**Figure 17:**
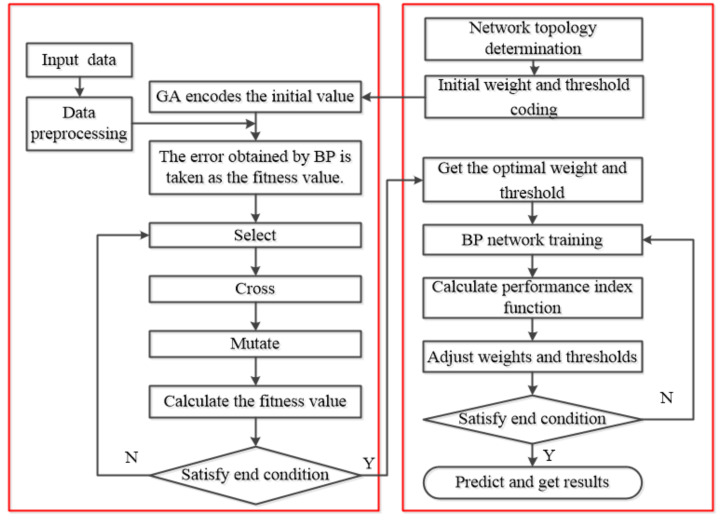
GA-BP algorithm flow chart.

The neural network optimized by genetic algorithm includes three parts [43,44]: BP neural network, genetic algorithm optimization and BP neural network prediction. In the BP neural network, the BP structure is determined according to the number of parameters of the fitting function, and then the length of the algorithm is defined. The path of the genetic algorithm is optimized by weight and threshold based on BP neural network optimization. Each individual in the population contains all weights and thresholds of the network. The adaptive value of the function is calculated by the individual fitness function. In the BP neural network prediction, the best individual is found through selection, crossover and mutation, and the initial value of the weight value and the threshold value is obtained through the genetic algorithm. The prediction output function is obtained after network training. The specific flowchart is shown in Figure 17. The structure of BP neural network is determined according to the number of input and output parameters of fitting function, and then the individual length of genetic algorithm is determined. The weight and threshold of the BP neural network were optimized by genetic algorithm. The adaptive value can be calculated by the adaptive function, and the genetic algorithm finds the optimum adaptive value corresponding to the individual by selecting, crossing and variation operation. The parameters of the optimal individual are assigned to the initial weights and thresholds of the network, and finally the prediction model after training the network is obtained.

The GA-BP neural network was built in MATLAB. The BP neural network was created by the *newff* function. The training function trainlm, and its training principle, is based on the Levenberg–Marquardt algorithm. For medium-sized BP neural networks, it has the fastest convergence speed. The maximum number of trains is 3000, and the convergence error is 0.001. The initial weight and threshold are the default value of the system. The parameter setting of the genetic algorithm is shown in Table 5. The adaptation function uses training data to train the BP neural network and predicts the prediction error of the training data as an individual adaptability value. After a series of selections, crosses, variations, and repetitive operations, the genetic algorithm can eventually find the best individual corresponding to the best adaptivity value. A function using a roulette method from a population was used to select an individual with ideal adaptability. Two individuals were selected by cross function from the overall, and new individuals are acquired at a certain probability. The variation function selects an individual from the population randomly and gets a new individual by a certain probability change.

We selected the sample input to punching speed, coefficient of friction, blank holder force, and specified the input layers X_1_-X_3_, respectively, with the node values of the three parameters. The output layer is the maximum thinning rate. 20 groups of 27 sets of data in Table 4 were selected as training data, and the remaining 7 groups were tested as test data.

**Figure 18:**
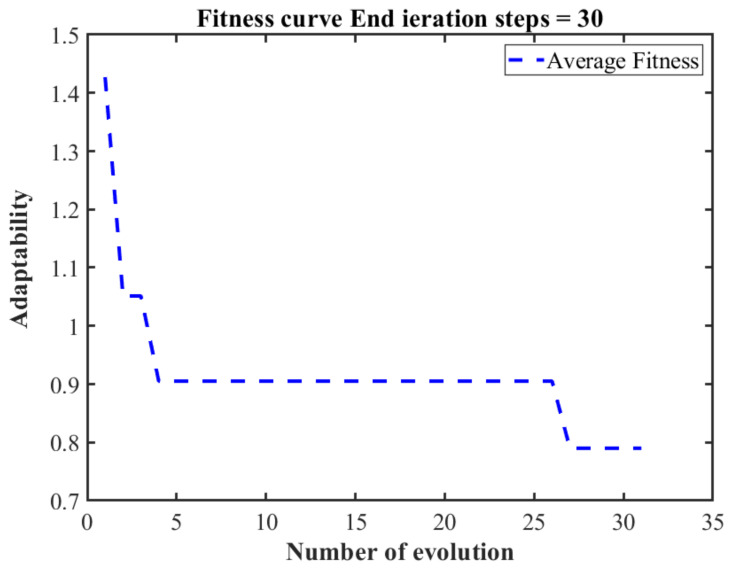
GA-BP Neural Network Application Curve.

Due to the quantitative level and unit of the punching speed, the coefficient of friction, and the blank holder force, the data must be prepared first. In order to improve the speed and convenience of the network computing and improve the prediction accuracy, the mapminmax function was used to normalize the data. Figure 18 shows the iterative process of adaptability in the genetic algorithm. It can be seen that when there is an iteration of approximately 27 times, the adaptivity value reaches the lowest.

**Figure 19:**
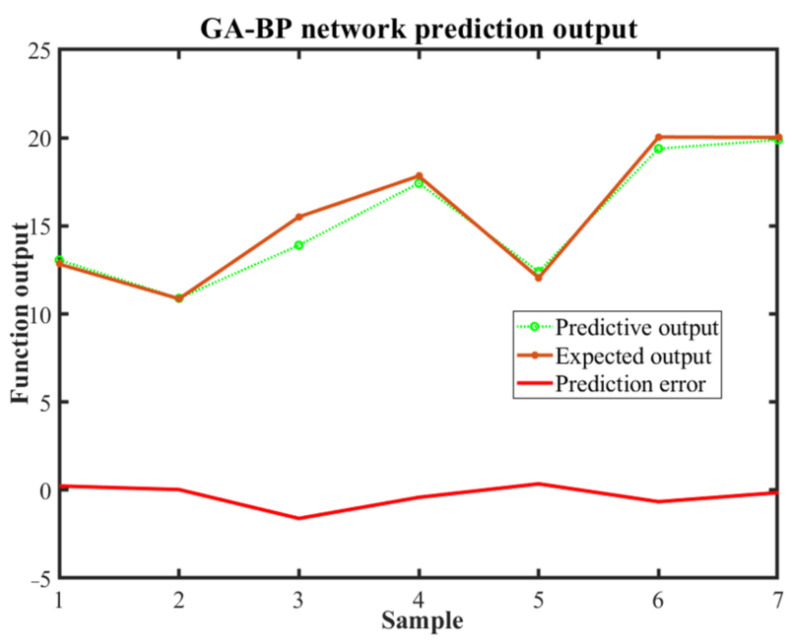
Comparison and error diagram of GA-BP neural network predictive value and expected value.

**Figure 20:**
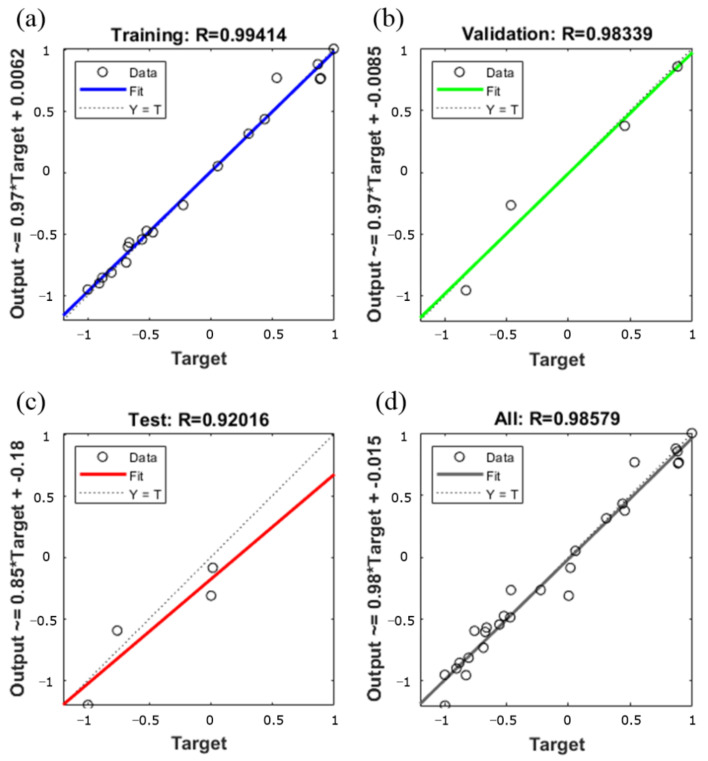
The results of multivariate regression analysis. (**a**) Training data (**b**) Validation data (**c**) Test data (**d**) All data.

Figure 19 is a comparative and error diagram of the GA-BP neural network prediction value and the expected value. From the figure, it can be seen that the prediction results of GA-BP neural network are accurate. The prediction error remains within 0.05. The results of the multiple regression analysis of the prediction results are shown in Figure 20. The correlation coefficient of the training data is 0.9941, indicating that the fit effect is very good. At the same time, the R values of regression analysis used to verify data and test data are 0.983 and 0.92, respectively, indicating that the model is reliable. The optimal process parameter of the 7075 aluminum alloy predicted by the GA-BP neural network is: the punching speed is 50 mm/s, the coefficient of friction between the die and the blank is 0.1 and the blank holder force is 5 kN. Predictive results: the maximum thinning rate is 9.37%.

### 4.4. Verification of Prediction Results and Finite Element Analysis

**Figure 21:**
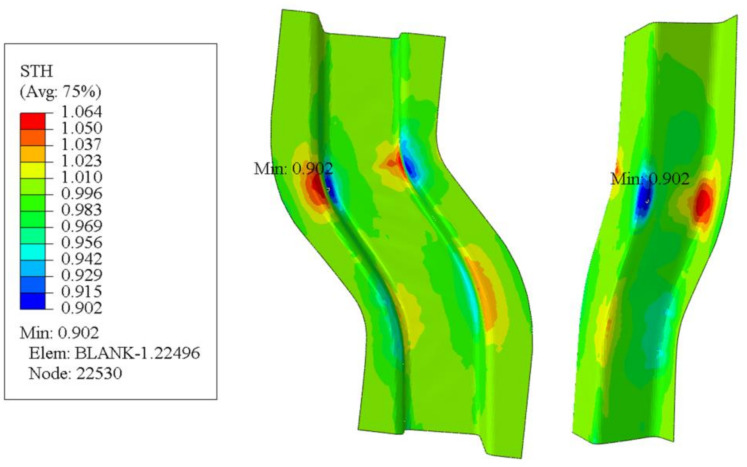
Thickness cloud map at stamping speed of 50 mm/s, friction coefficient of 0.1, and blank holder force of 5 kN, forming temperature 410 °C.

The process parameters predicted by the GA-BP neural network were entered into the Abaqus software, and the resulting S-rail thickness cloud map is shown in Figure 21. As can be seen from the figure, the minimum thickness of the S-rail is at the corner of the lower part of the left side wall, with a minimum thickness of 0.9019 mm. The maximum thinning rate of the S-track is 9.81%, and the error of the maximum thinning rate of 9.3% predicted by the GA-BP neural network is only 5%, which shows the accuracy of the prediction of the GA-BP neural network. It can also be seen in the thickness cloud map that the thickness distribution of the automotive S-rails formed using the process parameters predicted by the GA-BP neural network is relatively uniform, with only slight stacking at the corners of the upper part of the sidewalls.

**Figure 22:**
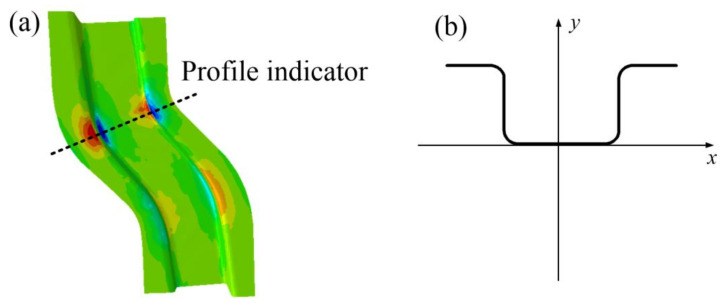
Schematic diagram of cross-sectional position (**a**,**b**) at optimal forming conditions (stamping speed of 50 mm/s, friction coefficient of 0.1, and blank holder force of 5 kN, forming temperature 410 °C).

**Figure 23:**
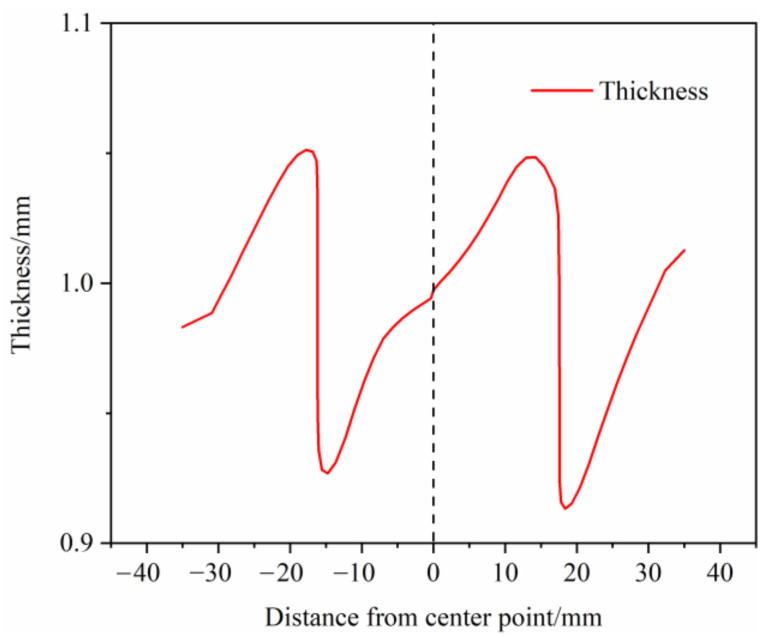
Thickness distribution at the profile for optimal forming conditions (stamping speed of 50 mm/s, friction coefficient of 0.1, and blank holder force of 5 kN, forming temperature 410 °C).

The forming result is taken in accordance with the cross-sectional indication line of Figure 22a and the thickness distribution of the S-rail is analyzed according to the layout of Figure 22b. Figure 23 shows the thickness distribution of the cross section. As can be seen in the figure, the thickness distribution in the flange of the S-rail and the bottom surface region is relatively uniform, approximately 1 mm. The stacker occurs at the corner where the left flange is connected to the sidewall and the bottom surface is connected to the right sidewall, and the thickness increases to 1.06 mm. At the corner where the left sidewall is connected to the bottom and the corner where the right flange is connected to the sidewall are thinned and the thickness is reduced to about 0.902 mm. The normalized average thickness and thickness standard deviation are calculated according to Equations (3) and (4). The normalized average thickness is 0.9913 mm and the standard deviation of thickness is 0.002 mm, indicating excellent hot stamping formability.

(3) $$\mathrm{NAT}=\frac{1}{nT_{\mathrm{ini}}}\sum_{1}^{n}T_{i}$$

(4) $$\mathrm{TD}=\frac{1}{n-1}\sum_{1}^{n}{\left(T_{i}-\mathrm{AT}\right)}^{2}$$

where, NAT is a normalized average thickness. *T*_ini_ is the thickness of the initial blank. *T_i_* is the thickness of *i* point. TD is the standard deviation of thickness values. AT is the average thickness of the formed part.

**Figure 24:**
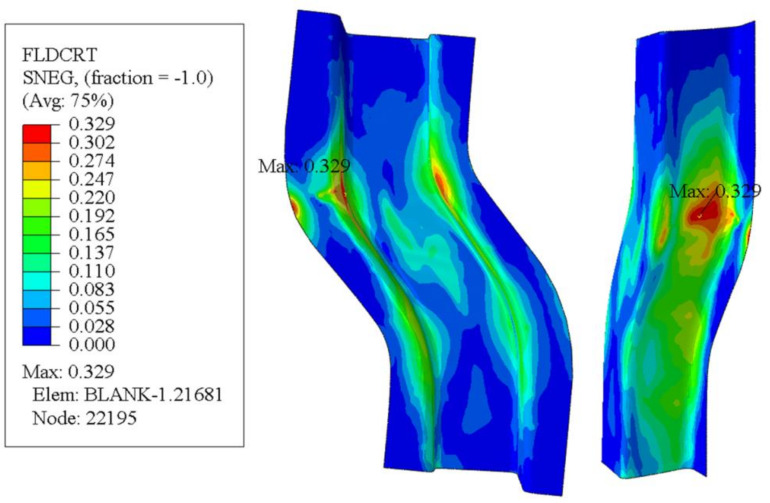
S-rail FLD damage cloud map at optimal forming conditions (stamping speed of 50 mm/s, friction coefficient of 0.1, and blank holder force of 5 kN, forming temperature 410 °C).

**Figure 25:**
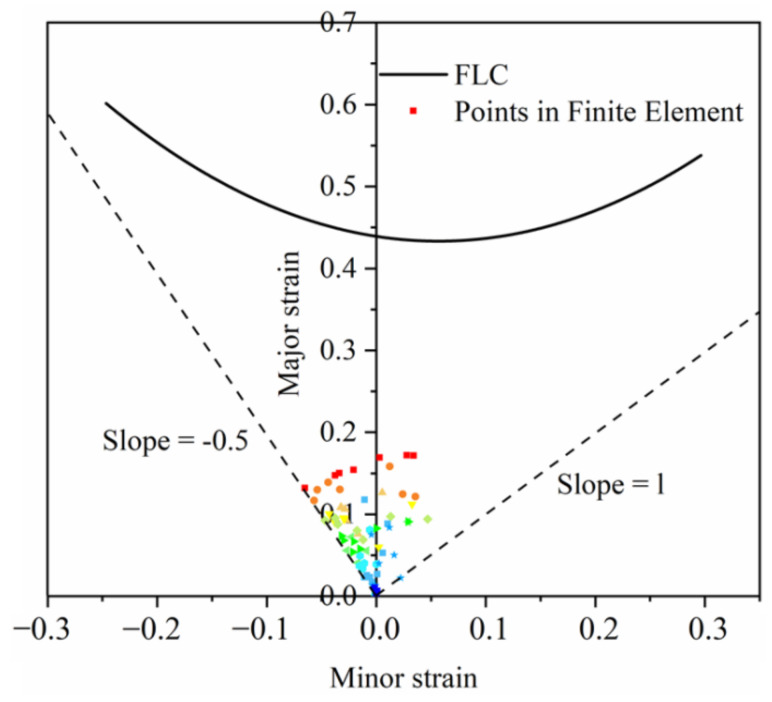
Forming limit result at optimal forming conditions (stamping speed of 50 mm/s, friction coefficient of 0.1, and blank holder force of 5 kN, forming temperature 410 °C).

Figure 24 is the damaged cloud map of the S-rail. As can be seen from the figure, the maximum damage occurs at the same location as the minimum thickness, which shows that the simulation is accurate. When the damage value reaches 1, the mesh is deleted, which means that the material fails to fracture. The maximum value of damage in Figure 24 is 0.3293, which indicates that only minor damage occurred to the material. The result of the finite element is randomly taken by 100 units of the major strain and the minor strain, as shown in Figure 25. It can be seen that all points are in the lower end of the FLC, the security zone [45]. Most of the points are to the left of FLC, which indicates that most of the automotive S-rails are in uniaxial tensile strain state and plane strain state during stamping, and only very few are in biaxial tensile strain state.

## 5. Conclusions

In this paper, 7075 aluminum alloy high temperature formability was studied. Mechanical properties under high temperature conditions of 7075 aluminum alloy are obtained by uniaxular thermal tensile experiments. The forming limit diagram of 7075 aluminum alloy was analyzed by high temperature Nakazima experiments, and the micro-morphologies of the fracture under different forming conditions were observed using SEM. Finally, numerical simulation and GA-BP neural network were used to optimize the stamping parameters of the hot-stamping automotive S-rails of 7075 aluminum alloy. The specific conclusions are as follows:

- In the isothermal forming limit experiment, when the stamping speed was 20 mm, the forming limit increased from 310 °C to 410 °C, and the maximum principal strain increased from 0.443 at 310 °C to 0.527 at 410 °C, an increase of about 14%. The change in stamping speed (10–40 mm/s) has a small effect on the forming limit of 7075 aluminum alloy within the applicable range of this experimental press.
- In the GA-BP neural network, to optimize the parameters of automotive S-rail hot stamping process, the results predicted by GA-BP are very close to the experimental results of the training samples. The correlation coefficient of training data reaches 0.9956, indicating that the fit effect is very good. Meanwhile, the R-values for the regression analysis used for the validation data and the test data reached 0.99873 and 0.9911, respectively, indicating that the model is reliable. The optimal process parameters for hot stamping of 7075 aluminum alloy predicted by GA-BP neural network are: stamping speed is 50 mm/s, friction coefficient between die and blank is 0.1, and the blank holder force is 5 kN. The maximum thinning rate is 9.37%.
- The hot stamping process parameters optimized by GA-BP neural network are input into ABAQUS for verification. The maximum thinning rate obtained is 9.81%, and the error with the maximum thinning rate predicted by the GA-BP neural network is only 5%, which illustrates the accuracy of the prediction of the GA-BP neural network. From the results, the normalized average thickness is 0.9913 mm, and the standard deviation of thickness is 0.002 mm. Comparing the results with the forming limit diagram, all units of the simulation results are in the safe zone, indicating excellent hot stamping formability.

## Figures and Tables

**Table 1:** Properties of 7075 aluminum alloy at room temperature.

Elongation (%)	Young’s Modulus (GPa)	Poisson’s Ratio	Vickers Hardness (HV)	Yield Strength (MPa)	Tensile Strength (MPa)
13	72	0.3	180	455	600

**Table 2:** Thermal properties of 7075 aluminum alloy.

Temperature (°C)	20	310	360	410
Thermal conductivity (W/(m·K))	121	985	1004	1023
Specific heat (/(kg·°C))	857	148	151	155

**Table 3:** Factor level table.

	Punching Speed (mm/s)	Coefficient of Friction	Blank Holder Force (kN)	Deformation Temperature (°C)
Level 1	10	0.1	1	410
Level 2	105	0.2	10.5	410
Level 3	200	0.3	20	410

**Table 4:** Orthogonal test scheme.

No.	Punching Speed (mm/s)	Coefficient of Friction	Blank Holder Force (kN)	Deformation Temperature (°C)	Maximum Thinning Rate (%)	The Data Type
1	10	0.1	1	410	33.08	Training data
2	10	0.1	10.5	410	30.44	Training data
3	10	0.1	20	410	35.83	Training data
4	10	0.2	1	410	35.3	Training data
5	10	0.2	10.5	410	31.65	Training data
6	10	0.2	20	410	37.15	Training data
7	10	0.3	1	410	36.14	Training data
8	10	0.3	10.5	410	33.48	Training data
9	10	0.3	20	410	39.32	Training data
10	105	0.1	1	410	20.63	Training data
11	105	0.1	10.5	410	18.98	Training data
12	105	0.1	20	410	22.36	Training data
13	105	0.2	1	410	23.74	Training data
14	105	0.2	10.5	410	20.06	Training data
15	105	0.2	20	410	27.32	Training data
16	105	0.3	1	410	25.05	Training data
17	105	0.3	10.5	410	21.1	Training data
18	105	0.3	20	410	30.85	Training data
19	200	0.1	1	410	25.33	Training data
20	200	0.1	10.5	410	22.05	Training data
21	200	0.1	20	410	27.06	Test data
22	200	0.2	1	410	26.82	Test data
23	200	0.2	10.5	410	23.08	Test data
24	200	0.2	20	410	28.06	Test data
25	200	0.3	1	410	27.93	Test data
26	200	0.3	10.5	410	24.38	Test data
27	200	0.3	20	410	29.14	Test data

**Table 5:** Parameters of genetic algorithm.

Program Name	Population Size	Number of Evolutions	Cross Probability	Variation Probability
Value	10	30	0.3	0.1

## Data Availability

Data is contained within the article. The data presented in this study are available in Research on High Temperature Stamping Forming Performance and Process Parameters Optimization of 7075 Aluminum Alloy.
